# Radiation therapy for lobular breast cancer: opportunities and challenges for leveraging radiosensitivity

**DOI:** 10.1038/s41523-025-00788-x

**Published:** 2025-07-10

**Authors:** Michael R. Boysen, Corey W. Speers, Matthew J. Sikora

**Affiliations:** 1 Department of Pathology, University of Colorado Anschutz Medical Campus, Aurora, CO USA; 2 University of Colorado School of Medicine, University of Colorado Anschutz Medical Campus, Aurora, CO USA; 3 Department of Radiation Oncology, University Hospitals Seidman Cancer Center, Cleveland, OH USA

**Keywords:** Radiotherapy, Cancer therapy, Breast cancer, DNA damage and repair

## Abstract

Invasive lobular carcinoma (ILC) is the most common special histological subtype of breast cancer, accounting for 15% of cases. ILC has unique clinical and molecular characteristics yet is treated largely agnostic of subtype. We explore challenges and opportunities in treating ILC, focusing on the underexplored sensitivity of ILC to ionizing radiation therapy (XRT). While ILC presents forms of resistance to chemotherapy and endocrine therapy, clinical data support that ILC have a distinct vulnerability to XRT, with XRT reducing recurrence rates in postsurgical contexts; molecular analyses identify putative defects in DNA repair in ILC that may underpin XRT sensitivity. However, gaps in the literature limit our understanding of XRT efficacy in ILC, and current treatment guidelines that inadequately address ILC-specific considerations limit XRT optimization. Future research should prioritize new clinical and mechanistic analyses of XRT efficacy, toward optimizing patient care and harnessing the full therapeutic potential of XRT for ILC.

## ILC is a biologically distinct subtype of breast cancer

Invasive lobular carcinoma (ILC) is the most common special histological subtype of breast cancer, versus the more common invasive ductal carcinoma (IDC, i.e., breast cancer of no special type), accounting for 15% of all breast cancer cases^1^. With approximately ~46,000 new cases in the U.S. annually, ILC as an independent cancer would be the 6^th^ most common cancer affecting women. Despite this prevalence, patients with ILC are effectively treated the same as other breast cancer patients, agnostic of histological subtype, but a growing understanding of the unique biology of ILC offers new opportunities for precision treatment and tailored clinical management.

Several recent reviews have discussed advances in our understanding of the unique clinical and molecular features of ILC^2–7^. In brief, ILC are primarily identified by a “single-file” pattern of tumor cell infiltration, associated with hallmark loss of cell adhesion factor E-cadherin (*CDH1*), distinguishing it from IDC where gland formation is present. ILC is predominantly hormone receptor-positive ( ~ 95% estrogen receptor α/ER-positive) and HER2-negative^8^, and are more frequently multicentric/multifocal than other breast cancers. Despite generally presenting with features associated with a favorable prognosis, due largely to ER positivity and low proliferation, clinical management of ILC presents unique challenges. For example, clinical and radiographic detection are confounded by ILC’s growth as single-file lines and sheets, more often leading to subtle breast thickening rather than a conspicuous mass as typically found in IDC. Consequently, patients with ILC typically present with larger tumors and a higher nodal burden than those diagnosed with IDC. Furthermore, ILC is more likely to present with subclinical nodal metastases compared to size and stage matched IDC^9^. Additionally, ILC is associated with a higher risk of long-term disease recurrence compared to IDC, regardless of ER status, with ≥30% increased risk of recurrence more than 5 years post-diagnosis versus IDC^10,11^. This increased risk of long-term recurrence is further complicated by a relatively limited benefit of chemotherapy for most patients with ILC^12–14^, and distinct forms of anti-estrogen resistance^7,15–17^, collectively limiting treatment options for advanced or metastatic ILC. Despite these links between ILC and therapy resistance, clinical and laboratory data reviewed herein suggest that ILC are uniquely responsive to ionizing radiation therapy (XRT). We discuss available data on the efficacy of XRT and why it may be underutilized in treating ILC, provide an overview of current considerations for the use of XRT for ILC, and discuss data from laboratory models of ILC that may underpin the distinct efficacy of XRT in this breast cancer type.

## Radiation therapy improves outcomes for patients with ILC

Outcome studies to date for ILC are from retrospective subset analyses, due to a lack of prospective consideration of tumor histology in most trials. As such, robust data on the benefit of radiation therapy for patients with ILC are limited and confounded by contrasting eras of therapy. These limitations manifest in two particular contexts. First, data that is stratified by XRT use (i.e., to assess the benefit of adding XRT) is limited to cohorts treated with mastectomy or with outdated breast-conservation strategies (discussed in this section), while most data is from studies of partial breast irradiation after breast conservation surgery (discussed in sections below). Second, available data are from primary breast cancer and adjuvant/post-surgical radiation therapy; we could not identify specific outcomes data for the treatment of metastatic ILC. This is consistent with recent analyses demonstrating an under-representation of ILC in trials for metastatic breast cancer^18,19^. Even with these limitations, there are several studies that confirm an overall benefit for XRT in reducing rates of recurrence for ILC, and suggest an outsized benefit for XRT for ILC relative to IDC. Table 1 summarizes referenced studies comparing outcomes with versus without XRT use, including their cohort characteristics, interventions, and key findings.

**Table 1:** ILC treated with XRT versus without XRT

Study	Cohort (ILC)	Cohort (IDC)	Intervention	Findings	Conclusions
**Diepenmaat et al.**^21^	383 ILC (170 PMRT, 213 only mastectomy)	0 IDC (ILC only)	Mastectomy; With versus without PMRT	• 5 & 8 year actuarial recurrence rate for ILC with PMRT were at 2.1% and 2.1%, versus mastectomy only at 8.7% and 9.5%, respectively (*p* = 0.018)	Local control is excellent for patients with ILC who received postmastectomy radiotherapy and significantly better than patients receiving no radiotherapy
**Fodor et al**.^22^	235 ILC (72 BCS [53 PBI, 19 NRT], 163 mastectomy [81 WBI, 82 NRT])	0 IDC (ILC only)	(BCS with PBI) or (Mastectomy with WBI) versus NRT	• 15 year DMFS and BCSS for BCS with PBI versus mastectomy with WBI were both 62% versus 70%, respectively (*p* = 0.2017, *p* = 0.1728) • 15 year actuarial rate of ipsilateral breast recurrence for BCS group was 10% with versus 53% without PBI; 16% with versus 13% without WBI for the mastectomy group, respectively (*p* < 0.0001, *p* = 0.3965)	Radiation provides a substantial survival benefit in patients with ILC
**Stecklein et al**.^20^	12703 ILC	86098 IDC	PMRT versus no PMRT; ILC versus IDC	•5 year BCSS in ILC improved from 80.9% without PMRT to 84.7% with PMRT (*p* = 0.003) •5 year BCSS in IDC improved from 71.4% without PMRT to 77.0% with PMRT (*p* < 0.0001)	PMRT significantly improves 5 year OS and BCSS for ILC patients to a comparable degree to that seen in IDC. PMRT appears to be significantly underutilized

*APBI* accelerated partial-breast irradiation, *WBI* whole-breast irradiation, *PMRT* post-mastectomy radiation therapy, *BCS* breast conserving surgery, *NRT* no radiation therapy, *IORT* intraoperative radiation therapy, *DMFS* distant metastasis free survival, *BCSS* breast cancer specific survival, *OS* overall survival.

A study by Stecklein et al. in 2016 reported that in The Surveillance, Epidemiology, and End Results (SEER) database, postmastectomy radiation therapy (PMRT) improved 5-year overall survival (OS) in ILC from 59.6 to 74.7% and in IDC from 56.3 to 69.8%, in patients that did not or did receive PMRT, respectively^20^. Moreover, PMRT improved 5-year breast cancer specific survival (BCSS) from 80.9 to 84.7% in ILC and 71.4 to 77.0% in IDC. Similarly, Diepenmaat et al.(2009) also analyzed the effects of PMRT therapy on ILC (absent a parallel IDC cohort), and noted a decreased actuarial risk of local recurrence in ILC from 8.7 to 2.1%, with versus without PMRT^21^. In 2011, Fodor et al. assessed the effects of postsurgical radiation therapy, as well as the impact of radiation therapy after breast conserving surgery (BCS). Specifically, radiation therapy after BCS decreased the ipsilateral recurrence rate from 53 to 10% in ILC, while the ipsilateral recurrence rate in the postmastectomy group was nearly the same at 16% with PMRT versus 13% without PMRT^22^. While more contemporary studies support that BCS and mastectomy provide equivalent outcomes for patients with ILC^23^, this study highlights that residual disease post-surgery for ILC is highly responsive to XRT, since in this older BCS cohort, recurrence with XRT was effectively equivalent to patients treated with mastectomy. However, Stecklein et al. noted that despite the benefit of PMRT, radiation was underutilized for patients with ILC or IDC, with <60% of eligible patients receiving PMRT regardless of histology^20^. Collectively, these studies highlight the underexplored yet promising role of radiation therapy in the management of ILC, given the improvements in survival rates and recurrence risks, and support that ILC are highly responsive to XRT.

## Treatment de-escalation with accelerated partial breast irradiation is evolving for ILC

Considering the survival benefit of PMRT for patients with ILC, the underutilization of PMRT raises important questions regarding the optimal approach to radiation therapy in ILC. Consistent with the high recurrence rates absent PMRT noted above and the elevated risk of long-term recurrence for ILC, ILC has an increased rate of locoregional recurrence post-mastectomy compared to IDC (18% vs 6% at 10 years^24^), highlighting the importance of XRT in this population. ILC treatment may be further confounded by higher rates of clinical and sub-clinical nodal involvement than IDC, limited benefit from neoadjuvant chemotherapy in down-staging disease, and some association with increased false negative rates on sentinel lymph node biopsy due to the diffuse growth pattern^9,10,25–28^. These characteristics suggest that PMRT may play an outsized role in reducing recurrence risk in patients with ILC^20^. However, as radiation therapy strategies evolve, de-escalation approaches such as partial breast irradiation (PBI) are increasingly feasible, particularly for patients with node-negative disease. This raises the question—can PBI provide adequate local control for ILC? Although PMRT is often indicated for patients with high nodal burden, patients with node-negative ILC may be considered for breast-conserving therapy (BCS) with PBI. This poses a complex challenge, as ILC’s multifocal and infiltrative growth pattern makes it less predictable and increases the risk of geographic miss with PBI, making precise targeting more difficult compared to IDC. The historical concerns surrounding geographic miss in ILC with PBI have contributed to the skepticism regarding the use of PBI in ILC. However, recent studies have shown improved outcomes with refined selection criteria and advanced radiation techniques, offering a more promising outlook for the use of PBI in treating ILC.

Historic evidence for the utility of radiation after breast conserving surgery relies on trials that treated women with whole breast radiotherapy (WBRT) after BCS, including the Early Breast Cancer Trialists’ Collaborative Group (EBCTCG) meta-analysis^29^. Though the EBCTCG study did not report tumor histology, this meta-analysis demonstrated a consistent benefit for local control with the addition of WBRT after surgery, with a 50-90% relative risk reduction of local recurrence after surgery with the addition of radiation. More recently, interest has shifted to treating even smaller volumes of the breast, via PBI and accelerated partial breast irradiation (APBI) targeting the lumpectomy cavity, to maintain tumor control yet reduce dose and toxicity to the normal breast tissue and surrounding organs. Recent advancements in APBI techniques have contributed to a notable reduction in local recurrence rates, challenging the previously held belief that APBI was inappropriate for women with ILC.

Modern APBI is characterized by enhanced precision in radiation delivery, primarily due to daily image guidance. This technological advancement ensures accurate targeting of the tumor bed and adjusts for day-to-day changes in patient positioning and anatomy, thus reducing the likelihood of radiation missing its intended target^30^. Moreover, the application of a 2 cm margin around the tumor bed, as opposed to narrower margins in earlier protocols, has significantly decreased the risk of missing microscopic disease, thereby improving local control^31^. Wider margin application in APBI may be particularly critical for ILC, which shows much higher rates of positive margins after surgical resection than other breast cancers^32,33^. Furthermore, the dosimetric properties of external beam radiation therapy (EBRT) commonly used in APBI features a slower dose fall-off compared to intra-operative PBI (IORT), ensuring that therapeutic doses extend sufficiently into the tumor margins, which is crucial given that tumor cells can reside a few millimeters from the visible tumor edges^31^. In contrast, IORT delivers a single high dose of radiation that extends only 1–5 mm from the tumor cavity, potentially missing microscopic disease that resides beyond this range, resulting in inadequate treatment of the peripheral tumor and higher local recurrence rates^34,35^. Technical enhancements in APBI — particularly the use of image guidance, expanded margins, and gentler dose gradients — have collectively contributed to increased efficacy in controlling local disease. Recent comparative studies have shown that updated APBI protocols provide equally effective, if not superior, local control versus IORT^36^. Collectively, the changing landscape of PBI methods must be taken into account when considering the suitability of PBI versus WBRT for the treatment of ILC.

## Understanding partial breast radiation efficacy for ILC is confounded by tumor features, patient selection, and evolving protocols

The underutilization of PMRT is not unique to ILC, yet as noted above the distinct growth pattern and multifocality of ILC creates unique barriers for the use of PBI following BCS. Recent studies have demonstrated that BCS followed by PBI can be equally effective to whole-breast irradiation (WBI) if patients are appropriately selected; referenced studies are summarized in Table 2. These studies center around patients treated with BCS, examining the comparative efficacy of PBI and WBI specifically in the setting of the less invasive surgery of BCS. For instance, one of the earliest examples of comparing the efficacy of PBI versus WBI was from the Christie Hospital study. Through comparing recurrence rates in PBI versus WBI, Ribeiro et al. found that ILC had a 5-year recurrence rate of up to 21% with PBI versus 5% with WBI^37^. Comparatively, IDC displayed a recurrence rate of 6.5% and 3% for partial breast and whole breast irradiation, respectively. In later follow-up on this cohort^38^, Ribeiro et al. found that recurrence rates at 8 years for ILC were 34% and 8% for PBI versus WBI, respectively, while the 8 year rates for IDC were 15% versus 11%, respectively. It is noteworthy that the high recurrence rates after PBI noted in ILC in this study suggest a likely “geographical miss” in appropriately treating these multifocal tumors with PBI; patients with ILC receiving PBI were not likely candidates by current guidelines, which unfavorably impacted the recurrence rate for PBI. However, the results illustrate that while ILC may be more difficult to treat with PBI due to its diffuse and multi-focal nature, the efficacy of XRT and radiosensitivity of ILC can be seen in the drastically decreased recurrence rates for WBI versus PBI in this study.

**Table 2:** ILC Treated with WBI versus APBI

Study	Cohort (ILC)	Cohort (IDC)	Intervention	Findings	Conclusions
**Ribeiro et al**.^37^	67 ILC (46 APBI, 31 WBI)	521 IDC (275 IDC APBI, 271 IDC WBI)	BCS; WBI versus APBI	• 5 year recurrence rate for ILC at 21% with APBI versus 5% with WBI • 5 year recurrence rate for IDC at 6.5% with APBI versus 3% with WBI in IDC	Poor choice of candidates for APBI unfavorably skewed results
**Ribeiro et al**.^38^	67 ILC (46 APBI, 31 WBI)	521 IDC (275 IDC APBI, 271 IDC WBI)	BCS; WBI versus APBI (follow up on 1990 study)	• 8 year recurrence rate for ILC at 34% with APBI and 8% with WBI • 8 year recurrence rate for IDC at 15% with APBI and 11% with WBI	If patient selection is performed appropriately, XRT is effective in treating ILC
**Orecchia et al**.^41^	ILC 110 (57 WBI, 53 APBI)	IDC 1038 (514 WBI, 524 APBI)	BCS; APBI with IORT versus WBI	• IBTR with IORT for ILC and IDC of 3.8% and 4.4% at 5 years, 7.6% and 8.3% at 10 years, and 12.6% and 13.6% at 15 years. • Overall IBTR for WBI of 0.5% at 5 years, 1.1% at 10 years, and 2.4% at 15 years.	IORT may be inferior to WBI for ILC and IDC, but IORT was similarly effective for ILC as IDC
**Levitin et al**.^39^	258 ILC (26 APBI, 232 WBI); Based on propensity score match: 52 ILC (26 APBI, 26 WBI)	0 IDC (ILC only)	BCS; APBI versus WBI	• Propensity score match found cohort of patients (*n* = 26) treated with WBI that was comparable to APBI	APBI is an effective alternative in well-selected patients with ILC
**Mills et al**.^43^	68 ILC	821 IDC	BCS + APBI; ILC versus IDC	• 10 year ipsilateral breast tumor recurrence for ILC and IDC was 14% and 2.5%, respectively • Evaluated existing criteria for APBI candidacy in ILC based on ABS, ASTRO and GEC-ESTRO guidelines	Patients deemed unsuitable or high-risk by the ABS, ASTRO and GEC-ESTRO were not at increased risk for ipsilateral breast tumor recurrence, suggesting that expansion of the current criteria should be considered.
**Braunstein et al**.^40^	132 ILC	0 IDC (ILC only)	APBI in ILC (Single intervention)	• 4 year local recurrence rate of 3.0% (95% CI: 0.56% - 9.5%) for ILC treated with APBI	APBI shows promising outcomes for ILC, consistent with invasive ductal carcinoma, but requires validation in larger, long-term trials.

*APBI* accelerated partial-breast irradiation, *WBI* whole-breast irradiation, *PMRT* post-mastectomy radiation therapy, *BCS* breast conserving surgery, *NRT* no radiation therapy, *IORT* intraoperative radiation therapy, *DMFS* distant metastasis free survival, *BCSS* breast cancer specific survival, *OS* overall survival, *ABS* American Brachytherapy Society, *ASTRO* American Society for Radiation Oncology, *GEC-ESTRO* The Groupe Europeen Curietherapie European Society for Radiotherapy and Oncology.

In contrast to these earlier studies performed in the 1990’s, more contemporary studies on PBI in ILC have shown less disparate outcomes compared to WBI, suggesting that PBI may be effective for ILC. In an abstract presented at the 2009 ASTRO Consensus, Levitin et al. compared APBI versus WBI for a cohort of patients with ILC (*n* = 26 vs *n* = 232, respectively), and reported no significant differences in outcomes with APBI versus WBI^39^. In 2024, Braunstein et al. assessed 132 patients with early stage T1-T2 ILC (*n* = 65) or mixed ILC/IDC (*n* = 67) treated with accelerated partial breast irradiation (APBI). These patients were found to have a 3% cumulative incidence of local recurrence^40^, contrasting with the results from the Christie Hospital study. Additionally, more recent studies on PBI with ILC often utilize additional measures in an attempt to both enhance efficacy and decrease negative co-morbidities, namely through the incorporation of intraoperative irradiation or brachytherapy. In the randomized, phase 3 equivalence trial on electron intraoperative radiotherapy (ELIOT), Orecchia et al. compared IORT versus traditional external beam WBI; the cohort included ILC (total *n* = 110; WBI, *n* = 57; IORT, *n* = 53) and IDC (total *n* = 1038; WBI, *n* = 514; IORT, *n* = 524)^41^. This study demonstrated ipsilateral breast tumor recurrence (IBTR) rates within the IORT group for ILC and IDC of 3.8% and 4.4% at 5 years, 7.6% and 8.3% at 10 years, and 12.6% and 13.6% at 15 years. Comparatively, external WBI had an overall IBTR (consisting of all breast cancer histologies) of 0.5% at 5 years, 1.1% at 10 years, and 2.4% at 15 years. Based on these findings, IORT is inferior to WBI for both ILC and IDC, yet IORT was not less effective for ILC versus IDC. In the German–Austrian APBI Phase II trial, Ott et al. found that there was no significant difference in outcomes between ILC (*n* = 45) and other breast cancer histologies (*n* = 229) when treating with PBI and brachytherapy, with 5-year local recurrence free survival of 97.6% and 97.8%, respectively^42^. Mills et al. also investigated the efficacy of PBI with brachytherapy, demonstrating that ipsilateral breast tumor recurrence rates with partial breast radiation and brachytherapy at 5 and 10 years were 1.3% and 2.5% for IDC, respectively, and 3.5% and 14% for ILC, respectively^43^. Importantly, the increased 10-year recurrence in ILC observed by Mills et al. may also be linked to the challenges associated with the diffuse nature of ILC and the application of APBI, but may also reflect the increased propensity of ILC for late recurrence and the longer-term follow up of this study. Despite that potential confounder, these observations taken together support that the initial findings from the Christie Hospital study on PBI for ILC may not reflect current opportunities for PBI for patients with ILC. Although previous studies have raised concerns about recurrence rates, recent investigations including intraoperative techniques and brachytherapy paint a more favorable picture. The importance of judicious patient selection emerges as a pivotal factor, as illustrated by the varied outcomes in the above studies.

## A lack of clear guidelines for radiotherapy use in ILC reflects gaps in the literature

The unique growth pattern of ILC is a major confounder for selecting XRT modalities in treating ILC, in particular for the application of clinical guidelines for XRT. For example, the American Society of Clinical Oncology (ASCO) and National Comprehensive Cancer Network (NCCN) guidelines lack specific recommendations for treating ILC with XRT, largely grouping ILC with other breast cancer histology when considering treatment. Current ASCO and ASTRO guidelines note the uncertainty regarding the magnitude of increased risk associated with breast cancers with unfavorable features such as ILC and therefore state that PBI is not recommended for any patients with lobular histology. Furthermore, most of the studies referenced in these guidelines specifically note that nearly all of the randomized controlled trials excluded patients with lobular histologies (i.e. the population treated with PBI represented less than 5% of enrolled patients), again limiting the interpretation of how current guidelines apply to ILC^44^. However, outright exclusion of PBI for patients with ILC may not be appropriate, since PBI presents with fewer side-effects and long-term risks. Opting for PBI offers a distinct advantage in avoiding the escalating levels of radiation exposure associated with WBI, presenting a compelling option for patients seeking to mitigate the negative impacts of treatment. Improved methods for patient selection are crucial to harness the potential advantages of PBI while minimizing associated risks^45^. However, currently available data for ILC is insufficient for effective treatment stratification.

Over the past 20 years, several groups have worked towards providing guidelines that could better predict when PBI would be indicated. In 2009, the Groupe Européen de Curiethérapie-European Society for Therapeutic Radiology and Oncology (GEC-ESTRO) provided recommendations on patient selection for APBI. From this work, they recommended three overall categories to guide patient selection for APBI - “APBI: (1) a low-risk group for whom APBI outside the context of a clinical trial is an acceptable treatment option; including patients ageing at least 50 years with unicentric, unifocal, pT1–2 (630 mm) pN0, *non-lobular invasive breast cancer* without the presence of an extensive intraductal component (EIC) and lympho-vascular invasion (LVI) and with negative surgical margins of at least 2 mm, (2) a high-risk group, for whom APBI is considered contraindicated; including patients ageing 64 years; having positive margins, and/or multicentric or large (>30 mm) tumors, and/or EIC positive or LVI positive tumors, and/or 4 or more positive lymph nodes or unknown axillary status (pNx), and (3) an intermediate-risk group, for whom APBI is considered acceptable only in the context of prospective clinical trials.” [emphasis added]^46^. GEC-ESTRO guidelines as such exclude lobular histology as a low-risk group eligible for APBI, yet it is unclear where lobular histology falls as intermediate or high risk.

In 2021, these recommendations were followed-up on and re-evaluated; Mills et al utilized the suitability criteria for APBI from the American Brachytherapy Society (ABS), American Society for Radiation Oncology (ASTRO), and GEC-ESTRO to perform a single institution retrospective review of 946 patients with invasive breast cancer (821 IDC cases and 68 ILC cases). From this study, it was determined that patients that had been deemed unsuitable or high risk by the combined guidelines were not at increased risk for ipsilateral breast tumor recurrence, again indicating that current selection criteria should be reevaluated. Additionally, none of the suitability criteria applied was able to predict patient relapse-free survival^43^. From these studies, the absence of effective selection criteria for ILC patients for PBI emphasizes the pressing need for tailored guidelines and accurate risk categorization specific to this subtype, as otherwise patients will be excluded from receiving PBI.

In 2024, the Assisi Think Tank Meeting (ATTM) on Breast Cancer assessed existing literature on the feasibility and ramifications to treating ILC with XRT^47^. ATTM concluded that there is insufficient evidence to justify distinct locoregional management practices for ILC compared to IDC in most scenarios. The potentially increased benefit of XRT for improving outcomes for patients with ILC versus IDC led to a recommendation that “ILC histology might be considered as a *relative* indication for PMRT”, while recommending caution in the use of PBI for ILC outside clinical trials, as currently noted in other guidelines. However, ATTM noted the lack of randomized-controlled trials for addressing any key questions about XRT efficacy, and no recommendations were linked to stronger levels of evidence from experimental or randomized clinical studies, also noting that most studies lacked key tumor biomarker data like ER status. ATTM emphasized the critical need for recording histological types in prospective trials and observational studies to enhance the evidence base and improve future patient care.

The current limitations in the guidelines regarding use of XRT for ILC underscore a critical gap in tailored recommendations for this specific breast cancer subtype. The broad categorization and lack of nuanced guidance for ILC results in a missed opportunity to optimize patient care. Moving forward, there is a pressing demand for new studies that can support more effective stratification guidelines for XRT use for patients with ILC.

## Scarcity of data on ILC radiation sensitivity and lab findings

With the growing appreciation of ILC as a distinct clinical entity among breast cancers, laboratory research on ILC is advancing in parallel to identify the molecular underpinnings of ILC biology^48,49^. Basic and translational studies in ILC point toward a unique phenotype in ILC cells that may ultimately mediate XRT sensitivity via distinct DNA damage response. DNA repair deficiencies that may indicate XRT sensitivity are typically linked to mutations in key DNA repair genes like *BRCA1*, *BRCA2*, *PALB2*, or related genes, yet these mutations are uncommon in ILC^50–52^. Moreover, ILC genomes typically lack the genomic scarring that otherwise defines “BRCAness”, suggesting that ILC are proficient in DNA repair. However, in the Cancer Genome Atlas, high-risk ILC were associated with an elevated “DNA damage response” signature in protein analyses^8^, potentially consistent with dysfunctional cellular signaling in response to DNA damage. Similarly, ILC carry a higher burden of tumor mutations than matched IDC^53,54^, which also progresses in metastatic ILC, despite the absence of “BRCAness” and related genomic features. These features together suggest that DNA repair may be dysfunctional in ILC, but the etiology and clinical implications of this potential DNA repair dysfunction are yet unknown.

Using laboratory models of ILC, our group has identified an ILC-specific interaction between estrogen receptor α (ER) and DNA repair machinery, which facilitates ER function yet compromises DNA repair^55,56^. This novel signaling mechanism limits the ability of cells to effectively initiate and propagate DNA repair. Notably, we showed that after treatment with XRT in vitro, IDC rapidly form intra-cellular ‘foci’ of the DNA repair protein RAD51, indicating the initiation of BRCA1/2-mediated DNA repair via homologous recombination (HR). In ILC cells, formation of RAD51 repair foci was markedly delayed, and ultimately ILC cells showed extremely limited resolution of DNA damage using specifically HR, paralleling a “BRCA-like” phenotype. These observations together suggest that ILC may present with a previously unappreciated form of DNA repair deficiency, which may underpin radiosensitivity. Identifying tumor biomarkers to understand the prevalence of this DNA repair dysfunction in ILC is important toward defining links between cellular phenotypes and clinical XRT efficacy. Moreover, given that nearly all patients will be treated with anti-estrogen therapy, understanding the interaction between ER and DNA repair capacity in ILC may yield important treatment combination strategies leveraging XRT sensitivity^56–58^.

## Conclusions

ILC presents unique clinical challenges among breast cancers, from its subtle manifestation making early detection difficult, to resistance to conventional treatments like chemotherapy and anti-estrogen therapy, compounded with increased risks of late recurrence. However, studies to date suggest that ILC are uniquely responsive to radiation therapy, which provides a particular benefit for patients in reducing risks of disease recurrence relative to other breast cancers. However, the limited and fragmented data from retrospective studies hinders a comprehensive understanding of the full potential of XRT for ILC. As a result, current treatment guidelines lack specificity for ILC, highlighting the critical need for new studies that can support more precise guidelines for patients with ILC. A nuanced strategy for selecting patients with ILC for radiation therapy, particularly in the realm of PBI, is essential. Importantly, a growing body of basic and translational research on ILC identifies a potential molecular foundation for radiation sensitivity, which may be leveraged clinically. Larger-scale studies and investigations into the mechanisms governing ILC’s response to radiation therapy are crucial, laying the groundwork for evidence-based guidelines that optimize patient care and establish a basis for more effective, targeted treatments.

## Methods

NCBI Pubmed, Google Scholar, and University of Colorado Anschutz Medical Campus Library article search engine were used to search for the terms “ILC radiation therapy”, “ILC partial-breast irradiation”, “ILC and radiation therapy outcomes”, “postmastectomy radiation therapy for ILC”, “ILC radiotherapy”, “accelerated partial breast irradiation in lobular carcinoma”. Searches were primarily conducted from October 2023 to December 2024.

## Data Availability

No datasets were generated or analysed during the current study.
